# 13-Methylberberine, a berberine analogue with stronger anti-adipogenic effects on mouse 3T3-L1 cells

**DOI:** 10.1038/srep38129

**Published:** 2016-12-05

**Authors:** Yit-Lai Chow, Mami Sogame, Fumihiko Sato

**Affiliations:** 1 Division of Integrated Life Science, Graduate School of Biostudies, Kyoto University, Kitashirakawa, Sakyo, Kyoto, 606-8502, Japan

## Abstract

Lipid metabolism modulation is a main focus of metabolic syndrome research, an area in which many natural and synthetic chemicals are constantly being screened for *in vitro* and *in vivo* activity. Berberine, a benzylisoquinoline plant alkaloid, has been extensively investigated for its anti-obesity effects and as a potential cholesterol and triglyceride-lowering drug. We screened 11 protoberberine and 2 benzophenanthridine alkaloids for their anti-adipogenic effects on 3T3-L1 adipocytes and found that 13-methylberberine exhibited the most potent activity. 13-Methylberberine down-regulated the expression of the main adipocyte differentiation transcription factors, peroxisome proliferator-activated receptor gamma (PPARγ) and CCAAT enhancer binding protein alpha (C/EBPα), as well as their target genes. PPARγ, C/EBPα, and sterol regulatory element binding protein 1 (SREBP-1) protein levels were reduced, and this lipid-reducing effect was attenuated by an AMP-activated protein kinase (AMPK) inhibitor, indicating that the effect of this compound requires the AMPK signaling pathway. Decreased Akt phosphorylation suggested reduced de novo lipid synthesis. C-13 methyl substitution of berberine increased its accumulation in treated cells, suggesting that 13-methylberberine has improved absorption and higher accumulation compared to berberine. Our findings suggest that 13-methylberberine has potential as an anti-obesity drug.

Alkaloids are naturally occurring, low molecular weight, nitrogenous secondary metabolites found in approximately 20% of flowering plant species. Many of these chemicals are biologically active, such as berberine, caffeine, colchicine, emetine, hyoscyamine, morphine, nicotine, and scopolamine^1^.

Berberine, a benzylisoquinoline alkaloid obtained from *Berberis* (*Berberidaccae*) and *Coptis* rhizomes, has traditionally been used to treat intestinal infections based on its antibacterial properties. In recent years, berberine has been reported to improve metabolic syndrome, leading to decreased plasma cholesterol and triglyceride levels in hypercholesterolemic patients and reduced body weight and plasma triglyceride levels, as well as improved insulin function in high-fat-fed rats, indicating potential as a new cholesterol-lowering drug^2^,^3^. We previously showed that benzylisoquinoline alkaloids modulated lipid metabolism in *Caenorhabditis elegans*^4^,^5^. In this study, we extrapolated our findings to a mammalian model. Using berberine as a reference compound, we screened protoberberine and benzophenanthridine alkaloids for effects on adipogenesis in 3T3-L1 mouse adipocytes. Among them, 13-methylberberine induced the most potent reduction in lipid accumulation. Thus, we further characterized the molecular mechanism of 13-methylberberine compared to berberine.

Adipocyte differentiation is a process that is tightly controlled by molecular and cellular mechanisms involving two main transcriptional factors, peroxisome proliferator-activated receptor gamma (PPARγ) and CCAAT enhancer binding protein alpha (C/EBPα)^6^. PPARγ is a member of the nuclear receptor superfamily of ligand-inducible transcription factors^7^ and is a master regulator of adipocyte differentiation and metabolism, controlling the gene networks involved in lipid metabolism and glucose homeostasis. C/EBPα is a transcriptional activator that functions in the differentiation process^8^. PPARγ and C/EBPα form a positive feedback loop to mutually sustain expression^9^, and together they regulate downstream target genes involved in adipogenesis. The phosphoinositide-3 kinase (PI3K)/Akt signaling pathway transduces the proadipogenic effects of insulin and promotes adipocyte differentiation by increasing PPARγ expression^10^.

Many studies have reported that PPARγ pathway inhibition and AMP-activated protein kinase (AMPK) pathway activation are the mechanisms by which berberine reduces lipid levels^3^,^11^,^12^,^13^. As 13-methylberberine is a close analogue of berberine, we examined its effects on PPARγ activation and on the upstream AMPK and Akt signaling. An alkyl substitution at position 13 enhanced the anti-adipogenic activity of 13-methylberberine; thus, we compared the structure-activity relationship of the benzylisoquinoline alkaloids in this study to berberine. The potential of 13-methylberberine as a candidate for metabolic syndrome treatment and its cytotoxicity are discussed.

## Results

### Effect of benzylisoquinoline alkaloids on lipid accumulation in 3T3-L1 adipocytes

**Figure 1:**
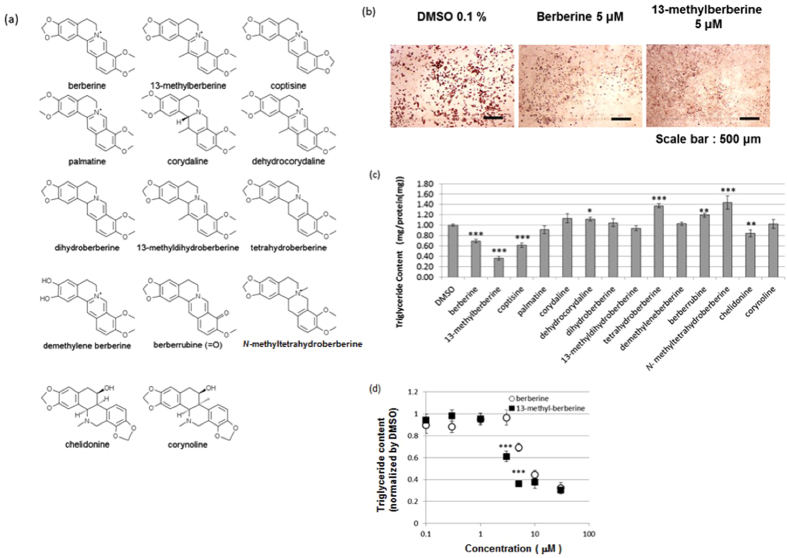
(**a**) Structures of the test compounds. (**b**) Oil Red O staining of 3T3-L1 adipocytes on Day 12. (**c**) Triglyceride content in 3T3-L1 adipocytes on Day 12; the results are normalized to the control (0.1% DMSO). All compounds were tested at 5 μM (containing 0.1% DMSO). n ≥ 9, error bar = SE. *p < 0.05, **p < 0.01, ***p < 0.001 two-tailed Student’s t-test. (**d**) Triglyceride content in 3T3-L1 adipocytes after treatment with various concentrations of berberine or 13-methylberberine. The results are normalized to the control (0.1% DMSO). n ≥ 6, error bar = SE. ***p < 0.001 two-tailed Student’s t-test.

We screened the anti-adipogenic effects of 11 protoberberine-type alkaloids (13-methylberberine, coptisine, palmatine, corydaline, dehydrocorydaline, dihydroberberine, 13-methyldihydroberberine, tetrahydroberberine, demethyleneberberine, berberrubine, and *N*-methyltetrahydroberberine) and 2 benzophenanthridine alkaloids (chelidonine and corynoline) (Fig. 1a) by treating differentiated 3T3-L1 adipocytes (Day 4) with 5 μM of each alkaloid. Purity of the alkaloids can be found in Supplementary Fig. S1. The adipocytes were stained with Oil Red O on Day 12. Among the treated cells, the 13-methylberberine-treated cells showed the lowest lipid droplet accumulation (Fig. 1b). Quantitative measurements of cellular triglyceride levels demonstrated that berberine, 13-methylberberine, coptisine, and chelidonine significantly reduced triglyceride accumulation, and 13-methylberberine had the most potent effect (Fig. 1c). The triglyceride-reducing effect of 13-methylberberine was dose-dependent from 0.3 to 10 μM. Moreover, 13-methylberberine exhibited a stronger effect than berberine at the same concentration (Fig. 1d).

### 13-Methylberberine inhibits adipogenesis and the expression of AMPK signaling pathway genes

We used RT2 Profiler PCR Arrays kits (QIAGEN) designed for pathway-focused gene expression analyses to characterize the molecular mechanisms by which 13-methylberberine and berberine exert their anti-adipogenic effects. We compared the expression profiles of genes involved in adipogenesis and the AMPK pathway in cells treated with berberine or 13-methylberberine with the non-treated control.

**Figure 2:**
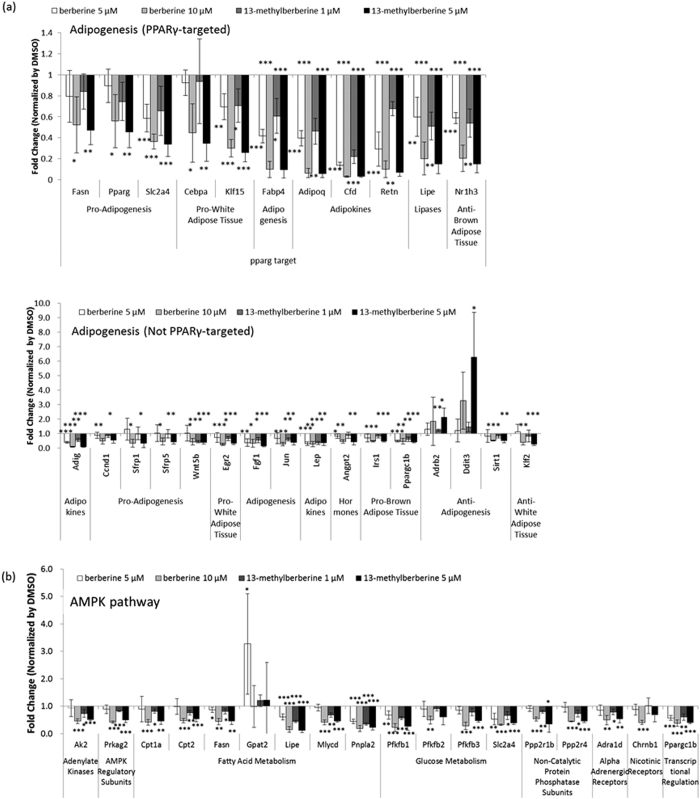
Effects of berberine or 13-methylberberine on the expression of (**a**) adipogenic pathway genes and (**b**) AMPK signaling pathway genes. 3T3-L1 adipocytes were treated with berberine or 13-methylberberine for 48 h. n = 3, error bar = SD. p < 0.05, **p < 0.01, ***p < 0.001 two-tailed Student’s t-test.

As shown in Fig. 2a, the expressions of many adipogenesis-related genes were significantly affected. The two main adipogenic transcription factors, PPARγ and C/EBPα, were significantly down-regulated, as well as some PPARγ target genes, including adipocyte-specific genes such as *Adipoq* (adiponectin), *Fabp4* (fatty acid binding protein 4), *Slc2a4* (glucose transporter member 4), and *Fasn* (fatty acid synthase). Alkaloid treatment suppressed the expression of genes involved in PPARγ signaling, particularly 13-methylberberine, which exhibited a strong effect at lower concentrations (5 μM) than berberine (10 μM). Furthermore, genes that inhibit adipogenesis, such as *Ddit3* (*Chop*) and *Adrb2*^14^,^15^,^16^, were dose-dependently up-regulated in 13-methylberberine-treated cells.

Likewise, many genes associated with AMPK signaling pathway were significantly down-regulated (Fig. 2b). Notably, 13-methylberberine and berberine treatment decreased the expression of fatty acid metabolism genes (*Fasn, Cpt1a, Cpt2, Lipe*, and *Mlycd*) and glucose metabolism genes (*Pfkfb1* and *Slc2a4*). Similar to the PPARγ signaling genes, 13-methylberberine more strongly down-regulated lipogenesis-related gene expression in the AMPK signaling pathway than berberine.

### Activation of AMPK signaling by 13-methylberberine

**Figure 3:**
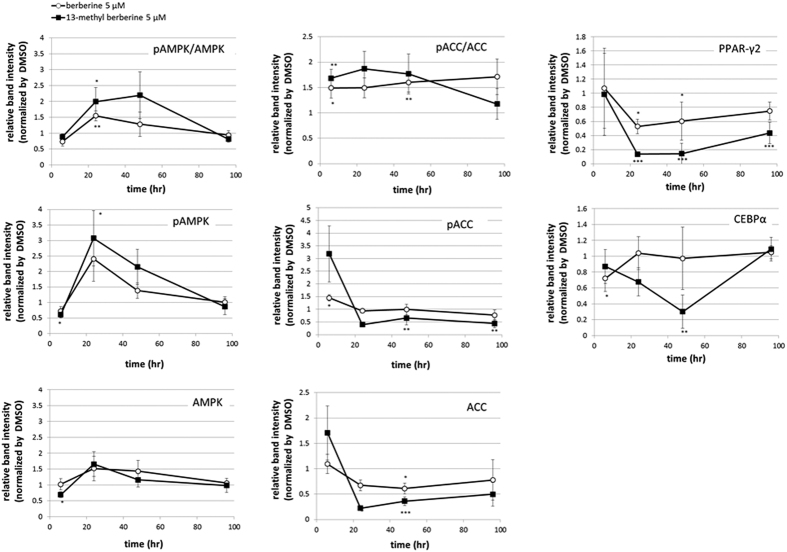
Immunoblot analyses showing effects of berberine- or 13-methylberberine-treated adipocytes on pAMPK/AMPK, pAMPK, AMPK, pACC/ACC, pACC, ACC, PPARγ2, and C/EBPα protein levels after 6, 24, 48 and 96 h. Values were normalized to control (DMSO). Band intensities were quantified using ImageJ. n = 6, error bar = SE. p < 0.05, **p < 0.01, ***p < 0.001 two-tailed Student’s t-test. (Immunoblot images can be found in Supplementary Fig. S5).

The lipid-reducing effect of berberine is reported to act via AMPK activation. Here, we examined the effects of 13-methylberberine and berberine on AMPK signaling components at the translational and transcriptional levels. The immunoblot analysis showed increased phosphorylation of AMPK and its downstream target, acetyl-CoA carboxylase (ACC), after alkaloid treatment (Figs 3 and S5), especially 13-methylberberine treatment. AMPK phosphorylation was observed after 24–48 h of treatment, and ACC phosphorylation was observed after 6–48 h of treatment. In our study, we found that ACC was phosphorylated earlier than AMPK. However, ACC protein levels were substantially decreased after the 24–96 h treatment, suggesting that both alkaloids affected lipid biosynthesis by increasing ACC phosphorylation and degradation to inhibit its activity.

13-Methylberberine treatment also significantly decreased PPARγ and C/EBPα levels after 24–48 h of treatment (Fig. 3), although berberine treatment had little effect on C/EBPα levels.

Quantitative real-time PCR and immunoblot analyses showed that both alkaloids affected the PPARγ and AMPK pathways in a similar manner. However, 13-methylberberine showed a stronger effect than berberine at the same concentration.

### 13-Methylberberine anti-adipogenic effects are dependent on AMPK signaling

**Figure 4:**
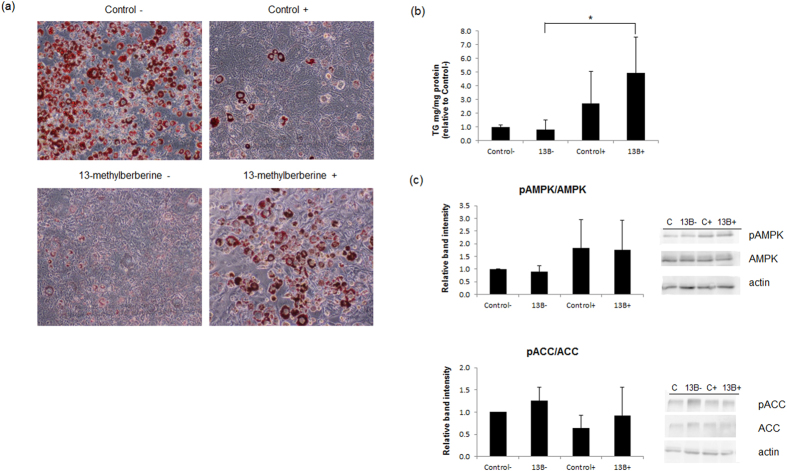
Effects of an AMPK inhibitor (Compound C) on control and 13-methylberberine-treated adipocytes. (**a**) Oil Red O staining in 3T3-L1 adipocytes on Day 12. n = 5 from 2 independent experiments. Images shown are results of a set of samples from one experiment. (**b**) Triglyceride content in 3T3-L1 adipocytes on Day 12. n = 6 from 2 independent experiments, error bar = standard deviation, *p < 0.05 two-tailed Student’s t-test. (**c**) Phosphorylation of AMPK and ACC at 6 h. n = 3 from 3 independent experiments, error bar = standard deviation. −/+ (without or with compound C), 13B–13-methylberberine 5 μM. The results are normalized to the control - (without Compound C).

Because our results showed that 13-methylberberine reduced the mRNA and protein levels of intermediates in the PPARγ and AMPK pathways, we investigated if its lipid-reducing activity required AMPK signaling. When adipocytes were co-treated with 13-methylberberine and compound C, an AMPK inhibitor, the lipid-reducing effect was suppressed. Lipid droplet accumulation was observed (Fig. 4a), and triglyceride levels were significantly increased (Fig. 4b) in cells co-treated with compound C and 13-methylberberine. Although AMPK phosphorylation was not affected by compound C treatment at 6 h, ACC phosphorylation level showed trend of decrease (Fig. 4c). Qualitative and quantitative triglyceride measurements indicated that AMPK inhibition by compound C attenuated the anti-adipogenic effect of 13-methylberberine on 3T3-L1 cells.

### 13-Methylberberine reduced SREBP-1 and AKT phosphorylation

**Figure 5:**
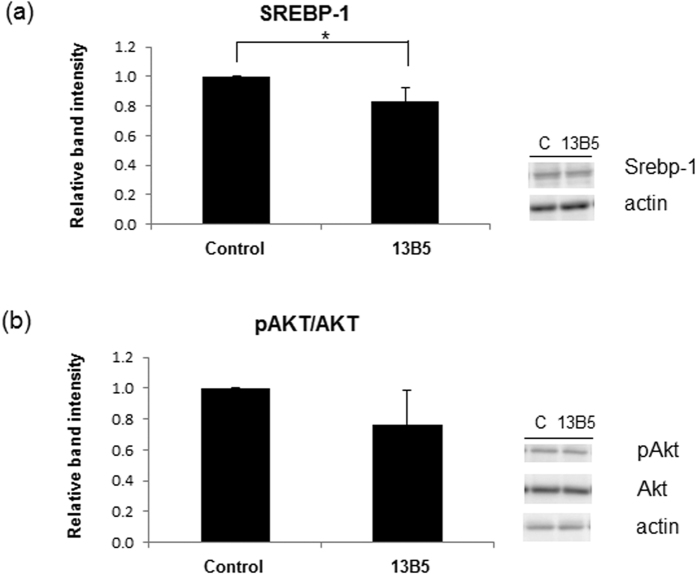
Effects of 13-methylberberine on (**a**) Srebp-1 and (**b**) Akt phosphorylation at 24 h. The results are normalized to the control. n = 3 from 3 independent experiments, error bar = standard deviation, *p < 0.05 two-tailed Student’s t-test. 13B5-13-methylberberine 5 µM.

Sterol regulatory element-binding proteins are transcription factors that regulate lipogenic gene expression. Carbohydrate response element binding protein (ChREBP) and SREBP-1c bind to the promoters of lipogenic genes, including ACC; FAS; fatty acid converting enzymes, such as stearoyl CoA desaturase; and fatty acid elongases, such as fatty acid elongase 6^17^. AMPK activation reduces ACC and FAS expression by down-regulating SREBP-1c. Activated AMPK directly inhibits ACC activity via phosphorylation and indirectly inhibits ACC expression by suppressing SREBP-1c^18^. Here, 13-methylberberine treatment reduced SREBP-1 levels in adipocytes (Fig. 5a), suggesting that lipid biosynthesis is strongly inhibited by direct phosphorylation of ACC and by decreased SREBP-1 levels, which both indirectly reduce ACC protein levels (Fig. 3).

Akt is involved in adipocyte differentiation, and its activation induces 3T3-L1 preadipocyte differentiation^19^,^20^. Akt also regulates the insulin signaling pathway in adipocytes. Insulin-stimulated Akt phosphorylation is critical for insulin-induced glucose metabolism, glucose transport, and adipocyte differentiation^21^,^22^. Akt reduction inhibited mouse embryonic fibroblasts differentiation into mature adipocytes^23^. The Akt signaling pathway activates PPARγ and C/EBPα during 3T3-L1 adipocyte differentiation, inducing adipogenesis. When we investigated Akt activation by analyzing Akt phosphorylation levels with immunoblots in control and 13-methylberberine-treated adipocytes, 13-methylberberine treatment showed trend of decreased phospho-Akt levels (Fig. 5b).

### *In vivo* accumulation and cytotoxicity of 13-methylberberine in 3T3-L1 cells

When we compared the effects of berberine and 13-methylberberine on adipogenesis, 13-methylberberine clearly exhibited stronger activity, including reduction in lipid droplet accumulation, triglyceride levels (Fig. 1b,d), and the mRNA (Fig. 2) and protein levels (Figs 3 and 5) of lipogenesis-related enzymes. AMPK was more strongly activated by 13-methylberberine treatment.

**Figure 6:**
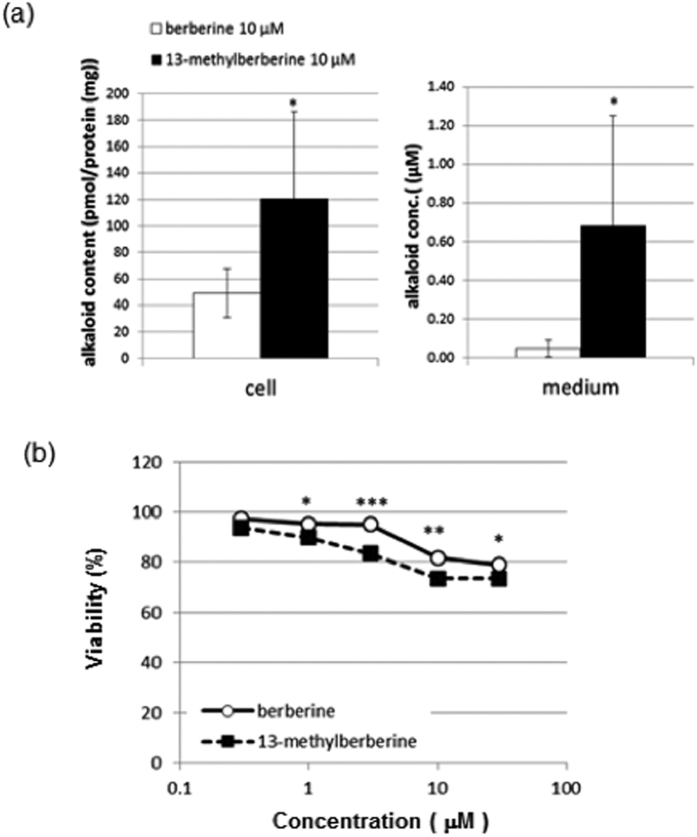
(**a**) Accumulation of berberine and 13-methylberberine in cells and medium after 48 h of treatment. n > = 6, error bar = SE. *p < 0.05 two-tailed Student’s t-test. (**b**) 3T3-L1 adipocyte viability after 24 h of treatment with various concentrations of berberine or 13-methylberberine. n ≥ 15, error bar = SE. *p < 0.05, **p < 0.01, ***p < 0.001 two-tailed Student’s t-test.

Next, we investigated the metabolic fate of both alkaloids in 3T3-L1 cells. Cells were treated with 10 μM berberine or 13-methylberberine for 48 h after which the cells and culture medium were collected for extraction of alkaloids and analysis using LC-MS. Studies have reported four main metabolites of berberine found in rat plasma: berberrubine (m/z 322), thalifendine (m/z 322), demethyleneberberine (m/z 324) and jatrorhizine (m/z 338)^24^. In our berberine-treated cell extracts, berberine (m/z 336) and m/z 338 were detected. In the 13-methylberberine-treated sample, only 13-methylberberine (m/z 350) was detected (Supplementary Fig. S2). In the cell culture medium extracts, berberine (m/z 336), m/z 284, 384, and 380 molecular ion peaks were detected, whereas 13-methylberberine (m/z 350), m/z 284, 366, 384, and 380 were detected in respective sample (Supplementary Fig. S3). We quantified the amount of berberine and 13-methylberberine using both alkaloids as standards. 49 pmole/mg protein of berberine and 121 pmole/mg protein of 13-methylberberine were detected in respective cell extract sample. In the cell culture medium, 0.048 μM berberine and 0.69 μM 13-methylberberine were detected (Fig. 6a). These results show 13-methylberberine accumulated at higher levels in the cell extracts and culture medium compared to berberine, suggesting that 13-methylberberine exhibits higher uptake and accumulation *in vivo*, which likely contributes to the higher potency of 13-methylberberine compared to berberine.

When cytotoxicity was evaluated, both alkaloids showed weak cytotoxicity in 3T3-L1 adipocytes. Treatment with 30 μM decreased cell viability by approximately 25% (Fig. 6b). However, the cytotoxicity of these alkaloids at the effective concentrations for lipid modulation was marginal and resulted in a 15 to 20% decrease in cell viability.

### Structure-activity relationship of the anti-adipogenic effects of benzylisoquinoline alkaloids

Only 3 (berberine, 13-methylberberine, and coptisine) of the 11 protoberberines tested in this study reduced triglyceride levels in 3T3-L1 adipocytes (Fig. 1c). Dihydroprotoberberines were less active than the oxidized form. 13-methyldihydroberberine only slightly reduced lipid accumulation, and dihydroberberine had no effect. A comparison of the methylene dioxy-ring-type and dimethoxy-type alkaloids indicated the importance of the methylene dioxy-ring for the lipid-reducing effect, because the 2–3 dimethoxy-type alkaloids palmatine, corydaline, and dehydrocorydaline showed no lipid reduction. Additionally, the loss of lipid-lowering activity in berberrubine indicated the importance of the 9-methoxy group.

Alkylation at position 8 or 13 of protoberberine alkaloids increases relative lipophilicity and substituent size and affects cytotoxic activity in cancer cell lines^25^,^26^. Derivatives of 13-substituted quaternary coptisine were more effective at inhibiting human tumor cell growth. These derivatives were suggested to have increased lipophilicity, which in turn enhanced absorption into bodily fluids^27^. In our study, C-13 methyl-substitution of berberine increased lipid-reducing activity, suggesting that the increased lipophilicity enhanced absorption into the cells. This result is consistent with the increased accumulation of 13-methylberberine in treated cells (Fig. 6a).

## Discussion

In this study, we examined the effects of several berberine analogues on adipogenesis in mouse 3T3-L1 cells. Oil Red O staining and triglyceride measurement results showed that 13-methylberberine treatment robustly inhibited lipid droplet accumulation. Further characterization of the molecular mechanism of the anti-adipogenic effect indicated that 13-methylberberine reduced the expression of the main adipogenic transcription factors, PPARγ and C/EBPα, at both the transcriptional and translational levels. qRT-PCR results showed the mRNA levels of PPARγ target genes and lipogenesis-related genes were more strongly suppressed in 13-methylberberine- than berberine-treated cells. We also investigated the involvement of AMPK and Akt signaling pathways, which are upstream of PPARγ and C/EBPα. Immunoblotting results showed that 13-methylberberine activated AMPK which directly reduced ACC levels via phosphorylation and indirectly via suppression of SREBP-1. ACC catalyzes the conversion of acetyl-CoA to malonyl-CoA, which is used in de novo fatty acid synthesis. Thus, reduction in ACC levels by 13-methylberberine inhibited lipid droplet accumulation in 3T3-L1 adipocytes. Furthermore, inhibition of AMPK by compound C attenuated the anti-adipogenic effect of 13-methylberberine, indicating that the lipid-reducing activity of 13-methylberberine is directly dependent on AMPK signaling. Akt signaling induces adipogenesis by activating the key transcription factors involved in the process, i.e. PPARγ and C/EBPα. Our immunoblot results demonstrated that Akt phosphorylation and activation were reduced by 13-methylberberine treatment, indicating that 13-methylberberine also modulates the Akt signaling pathway. All these results suggest 13-methylberberine acts on multiple molecular targets that contribute to its anti-adipogenic effect.

When we examined the cytotoxicity of berberine and 13-methylberberine, we found that the viability of 13-methylberberine-treated cells was slightly reduced compared to berberine at same concentration. However, the lipid-reducing activity of 13-methylberberine is stronger. When the amount of both alkaloids were quantified, higher concentration of 13-methylberberine was found in the cells and culture medium. The higher uptake and retention of 13-methylberberine *in vivo* could likely increase its potency compared to berberine.

An examination of the structure-activity relationship among the benzylisoquinoline alkaloids used in this study indicated the importance of the 2–3 methylene dioxy-ring, full oxidation of the protoberberine ring, and the methoxy residue at the 9 position in modulating 3T3-L1 adipocyte differentiation. These structural requirements would be useful for the development of new candidate chemicals with anti-adipogenic effects. Additionally, our investigation indicated the importance of methyl substitution at C-13 in enhancing uptake into the 3T3-L1 cells. The higher absorption and accumulation of 13-methylberberine in the cell contributed to its stronger activity at low doses compared to berberine.

Although it has not yet been clarified how these molecular structures affect lipid-reducing activity, berberine was reported to inhibit 3T3-L1 adipocytes differentiation through PPARγ pathways^28^. We found 13-methylberberine inhibited the PPARγ pathway more potently than berberine. Previous studies also suggested that berberine activates AMPK via AMP accumulation, which is induced by mitochondrial respiratory complex I inhibition in rats^29^. Although we have not performed experiments to determine the inhibitory activity of these chemicals on respiration, our finding that dihydroberberine, which inhibited respiratory complex I in rat, was ineffective in mouse 3T3-L1 cells suggests that further characterization of the action mechanism of berberine analogues is needed.

This study was aimed to screen plant benzylisoquinoline alkaloids for anti-adipogenic activity in 3T3-L1 cells which are extensively used to study adipogenesis and biochemistry of adipocytes. Our results indicated that 13-methylberberine inhibited adipogenesis more potently than berberine. This finding suggests 13-methylberberine is a potential drug lead for anti-obesity. Future studies using whole animal models would be crucial to evaluate its efficacy and safety for anti-obesity therapy.

## Materials and Methods

### Chemicals

Compound C was obtained from Sigma-Aldrich; dimethyl sulfoxide (DMSO) and all other chemicals were obtained from Wako Pure Chemical Industries, Ltd., unless indicated otherwise.

### Alkaloids

The alkaloids included berberine sulfide (Tokyo Chemical Industry Co., Ltd.), coptisine chloride, dehydrocorydaline nitrate (Wako), palmatine chloride (Mitsui Petrochemical Industries), 13-methylberberine chloride, corydaline, dihydroberberine, 13-methyldihydroberberine, tetrahydroberberine, *N*-methyltetrahydroberberine, chelidonine, corynoline (from Dr. Kinuko Iwasa, Kobe Pharmaceutical University), demethyleneberberine, and berberrubine (from Prof. Tomoo Hosoe, Hoshi University). The alkaloid samples were diluted in DMSO to a final concentration of 0.1% DMSO for the cell treatments. Purity of the alkaloids were analyzed by LC-MS (refer Supplementary Fig. S1).

### Antibodies

Phospho-Thr172-AMPK, AMPK, phospho-Ser79-ACC, ACC, PPARγ, C/EBPα, phospho-Thr308-Akt, and Akt were purchased from Cell Signaling Technology. Srebp-1 and Actin were purchased from Santa Cruz Biotechnology, and horseradish peroxide (HRP)-conjugated donkey anti-rabbit IgG was obtained from GE Healthcare.

### Cell culture

3T3-L1 cells range from Passage 9 to 11 (received from Dr. Masaya Nagao, Kyoto University) were cultured in Dulbecco’s Modified Eagle Medium (Wako) with 10% fetal bovine serum (Corning) at 37 °C and 5% CO_2_. Cells were passaged twice before used in assays to allow cells to re-establish normal cell cycle. Cell differentiation was induced at 2 days post-confluence (designated as Day 0) by adding 5 μg/mL insulin (Sigma), 500 μM isobutylmethylxanthine (Sigma), and 0.25 μM dexamethasone^30^ and cultured for two days. Subsequently, the cells were maintained in DMEM, 10% FBS and 5 μg/mL insulin and the medium was changed every two days. Alkaloids were added into the medium on Day 4 unless otherwise stated.

### Oil Red O staining

3T3-L1 adipocytes (Day 12) grown in cell culture plates were rinsed with phosphate buffered saline (PBS) and fixed in formalin for 30 min at room temperature. The formalin was removed, and the cells were rinsed twice with PBS. A 0.3% w/v Oil Red O (Sigma) solution was added at room temperature to stain the cells. After 1 h, the cells were rinsed in PBS twice, and lipid droplet accumulation was assessed under a microscope.

### Triglyceride and protein measurements

3T3-L1 adipocytes (Day 12) grown in cell culture plates were rinsed twice with PBS. Cell lysis buffer (1 M Tris-HCl pH 7.5, 1 M MgCl_2_, and 10% Triton X100) was added to each well, and the cells were harvested into an Eppendorf tube using a cell scraper. The cells were disrupted with an ultrasonicator, and the triglyceride and protein levels in the cell lysates were measured. The triglyceride concentrations were determined using the Triglyceride E test kit (Wako), and the absorbance was measured at 595 nm using a PowerScan4 (Biotek) plate reader. Protein concentration was determined using Bio-Rad DC Protein Assay (Bio-Rad) reagents, and the absorbance was measured at 595 nm using a PowerScan4 (Biotek) plate reader. The triglyceride content of each sample was normalized to its corresponding protein content.

### Gene expression analysis

3T3-L1 adipocytes (Day 4) were treated without or with alkaloids for 48 h and collected as described above. RNA was extracted using the RNeasy Mini Kit (QIAGEN). Reverse transcription was performed using 2 μg total RNA, and real-time PCR was performed using the RT^2^ Profiler PCR Arrays format D (QIAGEN) kit according to the manufacturer’s instructions. The data were analyzed using the ΔΔC_T_ method, and the relative transcript level was standardized using Gapdh as an internal control. The fold change between samples was normalized to the control (0.1% DMSO).

### Immunoblot analysis

3T3-L1 cells were rinsed twice with PBS. Cell lysis buffer (50 mM Tris-HCl, pH 7.4, 150 mM NaCl, 1 mM EDTA, 1% Triton X100, 0.1% SDS, 10 mM NaF, 1 mM Na_3_VO_4_, 50 mM Na_4_P_2_O_7_, and 1% protease inhibitor cocktail) was added to each well, and the cells were harvested into an Eppendorf tube using a cell scraper. The cells were disrupted using an ultrasonicator. The protein concentration in the cell lysate samples was measured as described above and adjusted to be equal with 2X sample buffer (0.1 M Tris-HCl, pH 6.8, 2% SDS, 12% β-mercaptoethanol, 20% glycerol, and 0.2% bromophenol blue). Twenty micrograms of protein were loaded onto the gel and separated by SDS-PAGE. The proteins were electro-transferred onto a polyvinylidene difluoride membrane (PVDF) (Millipore Immobilon-P) and probed with phospho-Thr172-AMPK, phospho-Ser79-ACC, phospho-Thr308-Akt, PPARγ, C/EBPα or Srebp-1 antibodies in 5% BSA/Tris-buffered saline with Tween-20 (TBST), followed by an HRP-conjugated secondary donkey anti-rabbit IgG in 5%BSA/TBST. Chemiluminescence was detected using the ChemiDoc Touch imaging system (Bio-Rad). The blots were then stripped and reprobed with AMPK, ACC, Akt or actin antibodies and detected with chemiluminescence. The intensity of the target protein band was quantified using ImageJ software (NIH).

### AMPK inhibitor assay

3T3-L1 adipocytes (Day 4) were pretreated with 10 μM compound C for 1 h. Alkaloids were then added and incubated with the cells for an additional 6 h. The cells were collected and lysed as described above.

### Alkaloid accumulation analysis

3T3-L1 adipocytes (Day 4) were treated with alkaloids (or no treatment for control) for 48 h, and protein samples were collected. Cell lysates and cell culture media were extracted with methanol using a Sep Pak C18 cartridge (Millipore). The methanol extracts were concentrated using a rotary evaporator. The samples were diluted in DMSO and injected into an LCMS2020 system (Shimadzu) using a TSK-gel ODS-80T_M_ 4.6 × 250 mm column, a column temperature of 40 °C, a sample volume of 10 μL, a flow rate of 0.5 mL/min, and a 0–15 min AcCN:H_2_O = 40:60, 18–40 min AcCN:H_2_O = 80:20, 40–50 min AcCN:H_2_O = 40:60 (containing 1% CH_3_COOH) gradient in positive SIM-SCAN mode. The scan mode ranged from m/z 200–700 and 10–500, and the SIM mode ranged from m/z 272–398.

Alkaloid concentrations were calculated based on LC peak area at 280 nm relative to standards peak area and those values were normalized to the protein content in cell extracts. (Supplementary Fig. S4).

### Cytotoxicity assay

3T3-L1 cells were cultured at density of 1.5 × 10^4^ cells/mL in 96-well plates for 16 h. The culture medium was then replaced with fresh medium, treated with alkaloids, and cultured for an additional 24 h. Cell viability was determined using Cell Counting Kit-8 (Dojinbo). The cells were incubated with the reagent for 1 h, and the absorbance of the living cells was measured using a PowerScan4 (Biotek) plate reader at 450 nm.

## Additional Information

**How to cite this article**: Chow, Y.-L. *et al*. 13-Methylberberine, a berberine analog with stronger anti-adipogenic effects on mouse 3T3-L1 cells. *Sci. Rep.* **6**, 38129; doi: 10.1038/srep38129 (2016).

**Publisher's note:** Springer Nature remains neutral with regard to jurisdictional claims in published maps and institutional affiliations.

## Supplementary Material

Supplementary Figures
