# Supplementary material for: 13-Methylberberine, a berberine analogue with stronger anti-adipogenic effects on mouse 3T3-L1 cells
Source: Sci Rep. 2016 Dec 5;6:38129. doi: 10.1038/srep38129 (PMC5137028; doi:10.1038/srep38129)
Supplement: Supplementary Figures [file srep38129-s1.pdf]

Supplementary Information

13-Methylberberine, a berberine analog with stronger anti-adipogenic effects on mouse 3T3-L1 cells

Yit-Lai Chow[1,2], Mami Sogame[1,2] and Fumihiko Sato[1]*

[1]Division of Integrated Life Science, Graduate School of Biostudies, Kyoto University, Kitashirakawa, Sakyo, Kyoto 606-8502, Japan

Supplementary Fig. S1

LC chromatogram — % Purity

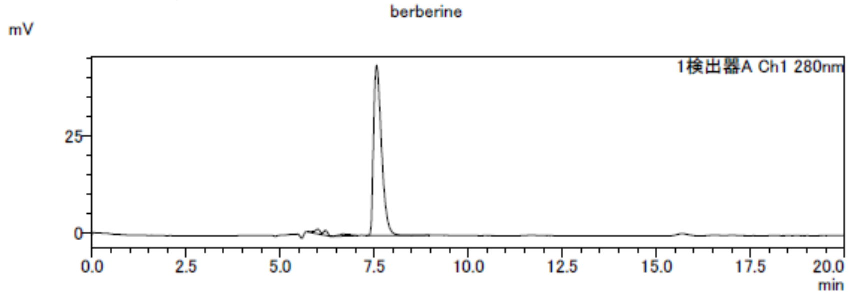

98.3

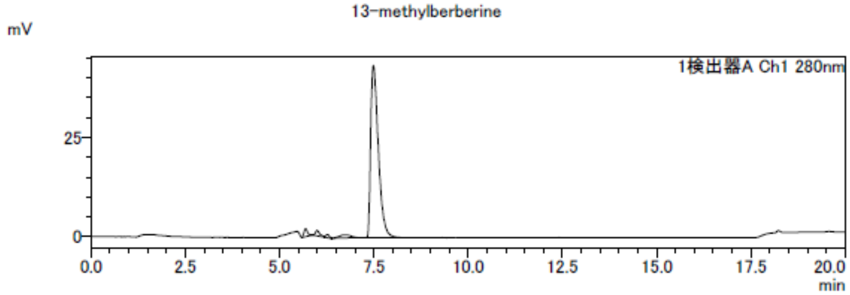

97.2

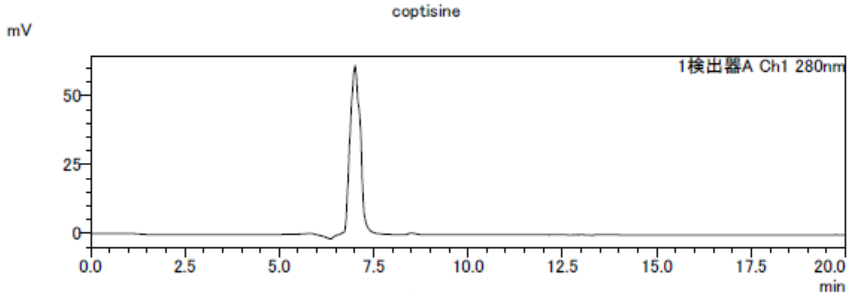

99.3

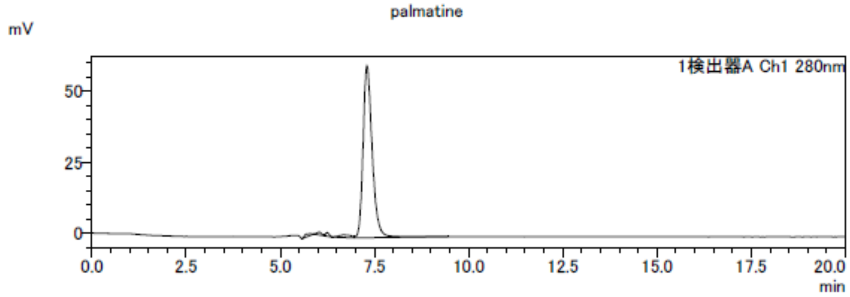

98.0

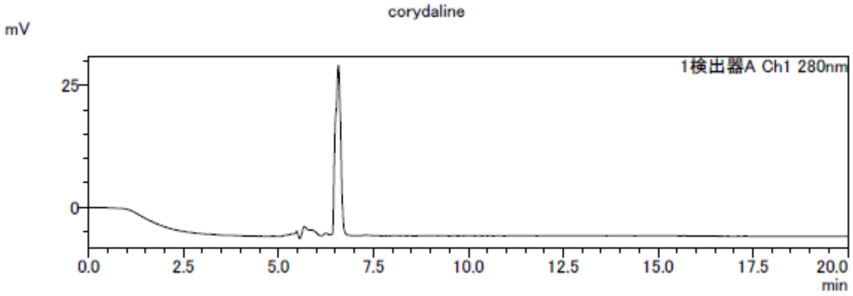

99.2

LC chromatogram — % Purity

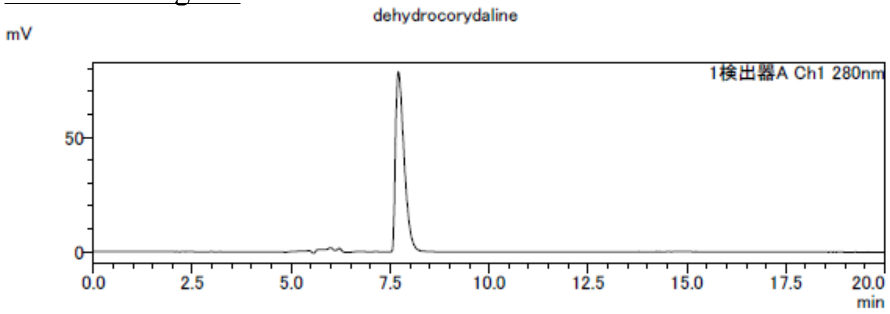

99.0

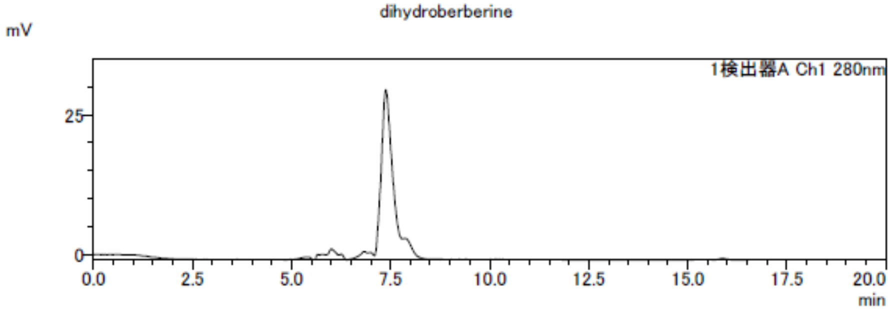

97.7

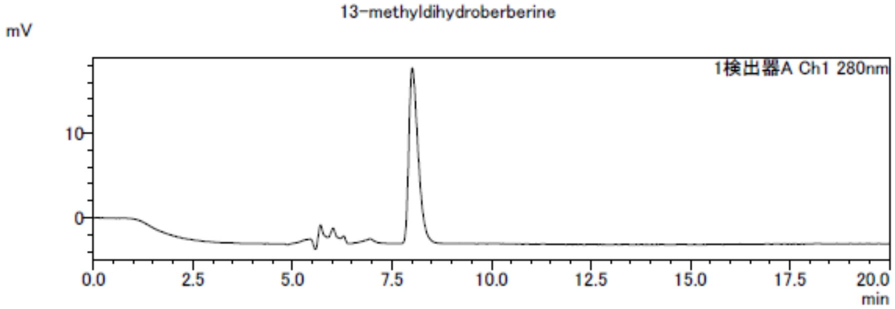

97.1

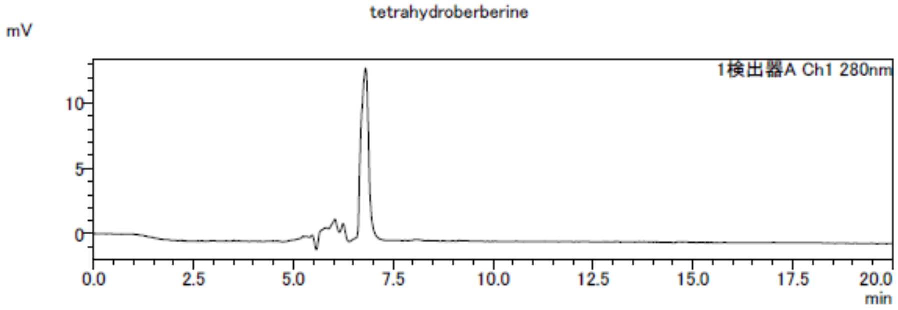

96.0

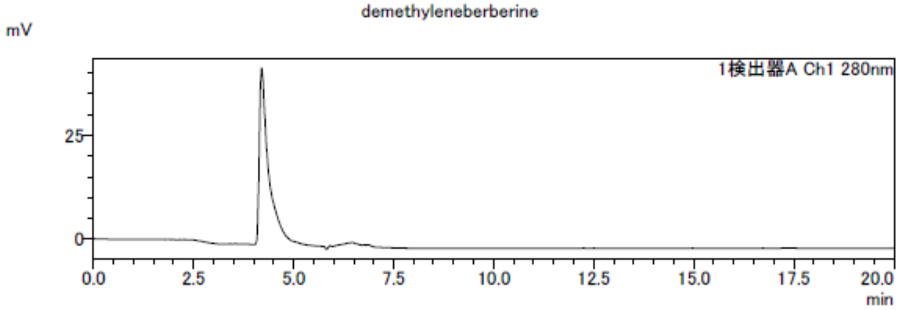

97.6

LC chromatogram % Purity

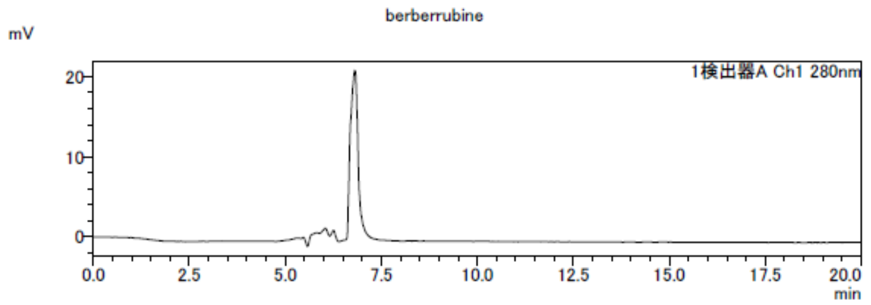

96.1

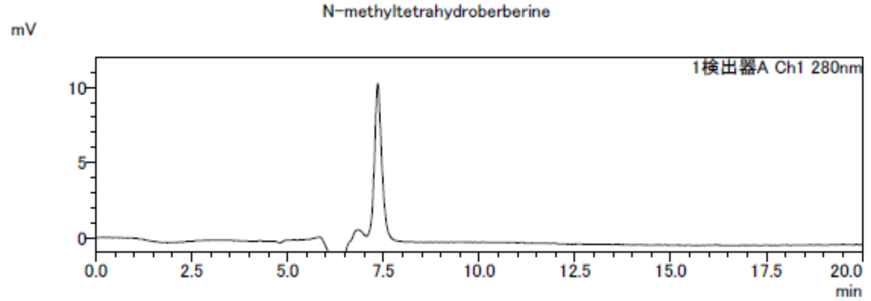

99.7

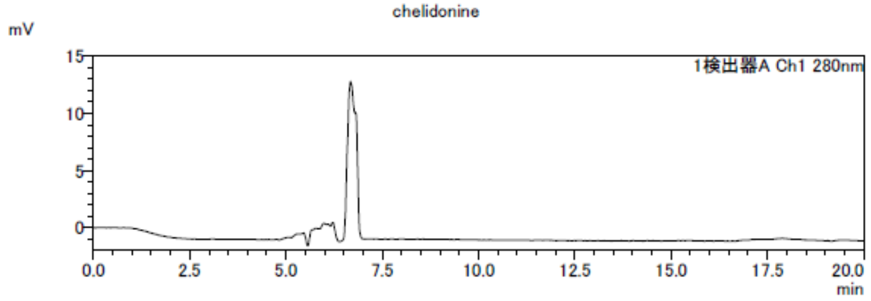

98.3

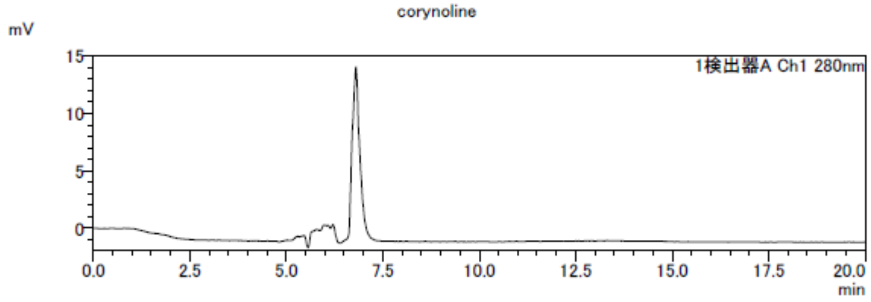

98.5

LC chromatograms of alkaloids used in this study measured at UV absorbance 280 nm. Purity is calculated based on the % peak area of each alkaloid.

Method: LCMS2020 system (Shimadzu) using a TSK-gel ODS-80Ts 4.6 x 250 mm column. Column temperature of 40°C; flow rate of 0.5 mL/min, and a 0-15 min AcCN:H2O=40:60, 18-20 min AcCN:H2O=80:20 (containing 1% CH3COOH) gradient in positive SIM-SCAN mode. The scan mode and SIM mode ranged from m/z 300-400.

Supplementary Fig. S2

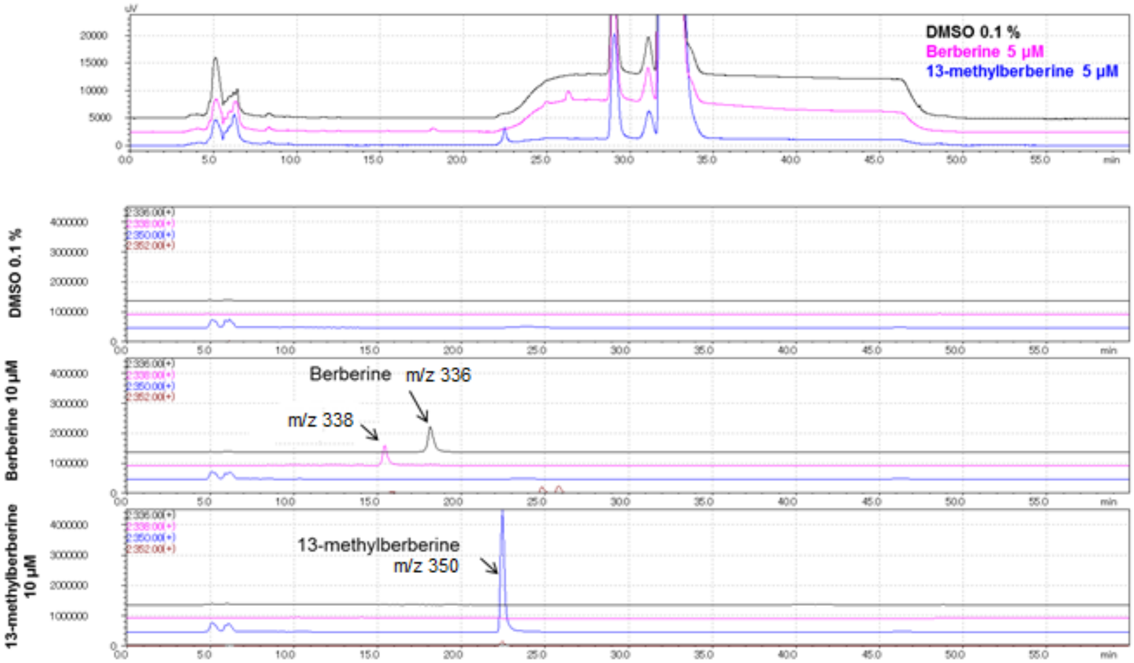

LC and MS chromatograms of cell extracts from 0.1% DMSO-, 10 μM berberine- and 10 μM 13-methylberberine-treated samples.

Supplementary Fig. S3

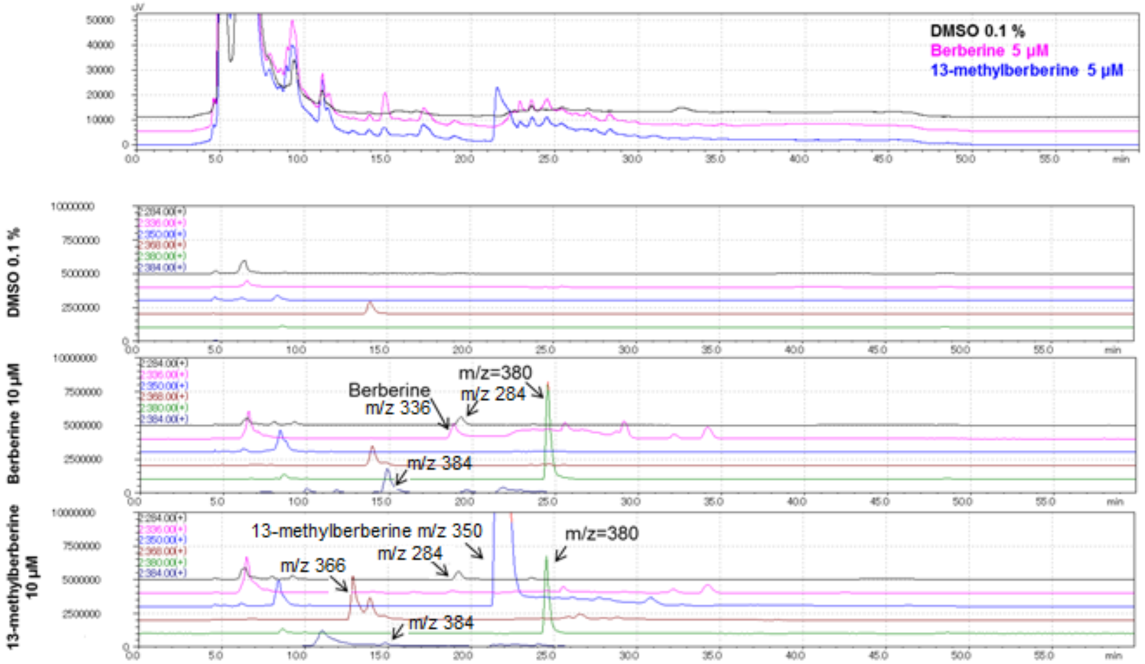

LC and MS chromatograms of culture medium extracts from 0.1% DMSO-, 10 µM berberine- and 10 µM 13-methylberberine-treated samples.

Supplementary Fig. S4

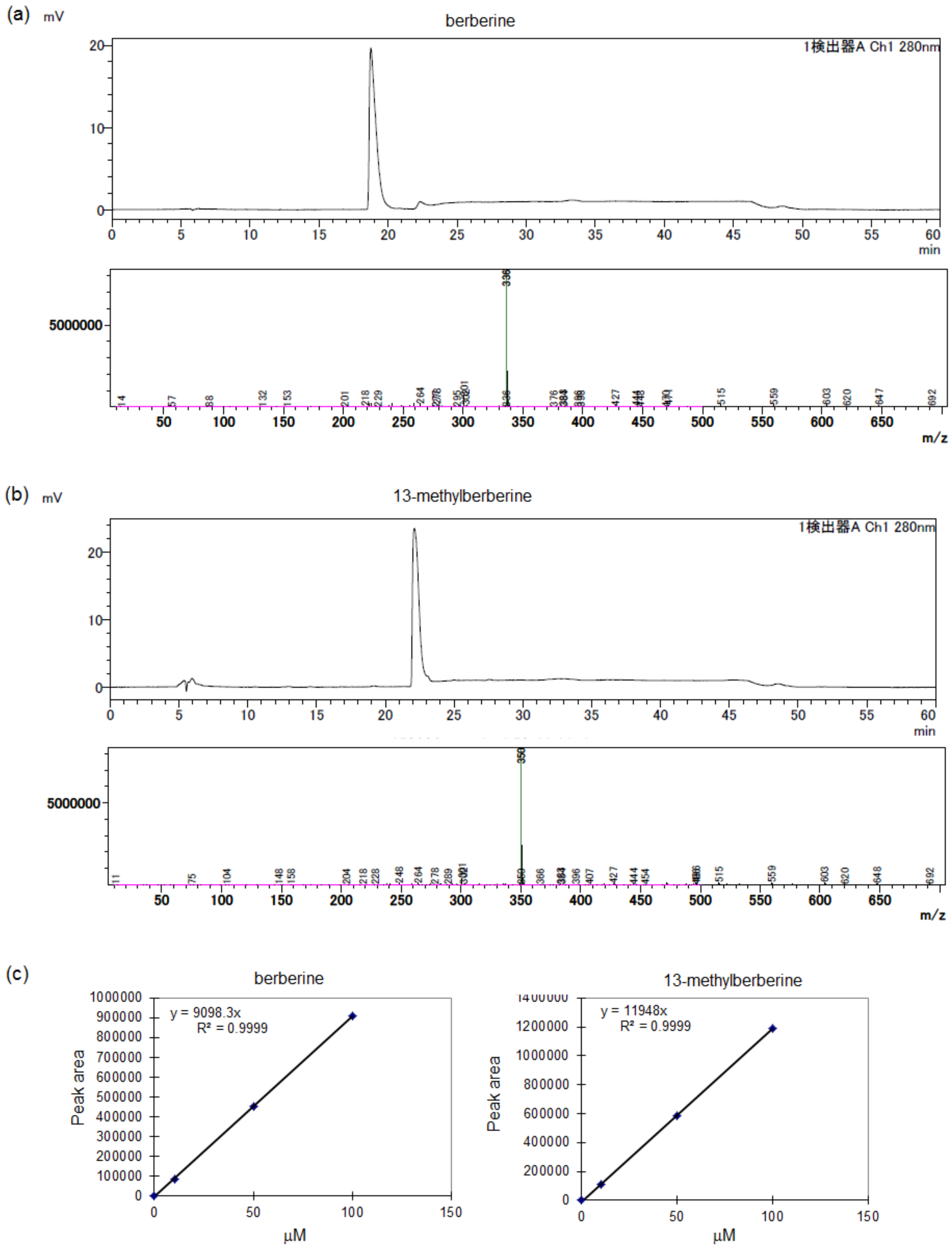

LC chromatogram and mass spectra of (a) berberine (m/z 336) and (b)13-methylberberine (m/z 350); (c) calibration curve of berberine and 13-methylberberine standards.

Supplementary Fig. S5

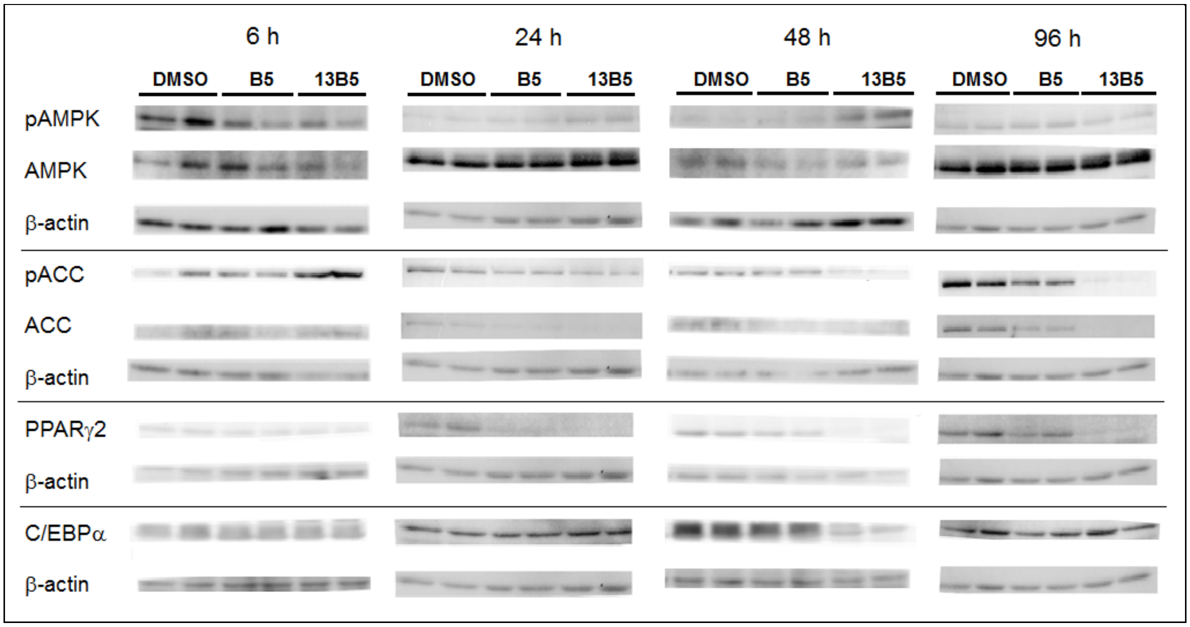

Immunoblot analyses showing bands of pAMPK, AMPK, pACC, ACC, PPARγ2, C/EBPα, and β-actin levels of control (DMSO), 5 μM berberine (B5)- or 5 μM 13-methylberberine (13B5)-treated adipocytes after 6, 24, 48, and 96 h.
